# Unlocking the circular potential: A review and research agenda for remanufacturing in the European wood products industry

**DOI:** 10.1016/j.heliyon.2024.e40264

**Published:** 2024-11-12

**Authors:** Mirka Kans, Malin Löfving

**Affiliations:** a Department of Technology Management and Economics, Division of Supply and Operations Management, Chalmers University of Technology, Vera Sandbergs Allé 8, 412 96, Gothenburg, Sweden; b School of Engineering, Department of Product, Production and Design, Jönköping University, Box 1026, 551 11, Jönköping, Sweden

**Keywords:** Remanufacturing, Wood products industry, Literature review, Research agenda, Europe, Circular strategies

## Abstract

While circularity has gained significant attention in recent years, the wood products industry remains an understudied sector in terms of remanufacturing practices. This study addresses this research gap by synthesizing the existing research on remanufacturing in the wood products industry and developing a research agenda tailored to the European context based on a structured literature review. Content and thematic analyses of peer-reviewed publications founded the basis of the synthesis. Three distinct perspectives on remanufacturing in the wood products industry emerged from the analysis: systems, customer, and manufacturer. These perspectives provide a framework for understanding the complex dynamics of remanufacturing in this sector. The proposed research agenda for the European context is intended to stimulate and guide future research efforts. A central priority identified is the need for enhanced collaboration and communication among actors within the value chain. Additionally, the importance of gaining a deeper understanding of customer perceptions and behavior in the context of remanufactured wood products is recognized. The development of economically viable remanufacturing strategies is another key focus. This knowledge is crucial for tailoring products to meet consumer needs and preferences, ultimately driving demand for remanufactured items. In conclusion, remanufacturing in the wood products industry is an area ripe for exploration, and this study represents a critical step towards catalyzing the research efforts necessary for its advancement.

## 1 Introduction

Remanufacturing, the process of refurbishing used products on an industrial scale, has gained increased attention recently [1]. Previous review articles covered various aspects of remanufacturing. These include technologies for remanufacturing [2,3], planning and decision-making aspects [4,5], quality, reliability, and maintenance aspects of remanufacturing [6], pricing strategies and competitive dynamics [7], as well as investigations into customer behavior concerning remanufactured products [8]. Salah et al. [1] noted the evolution of remanufacturing research, with a significant increase in articles post-2010, surpassing 350 annually in 2019 and 2020, and interest is still increasing.

Remanufacturing has successfully been implemented in various sectors, including electrical products [[9], [10], [11], [12]], heavy equipment [6,[13], [14], [15]], and in the automotive industry [16,17]. However, industries such as healthcare and furniture, despite high potential, have not fully embraced remanufacturing practices [18]. Notably, the furniture industry is a vital component of the wood products sector, encompassing a wide range of value-added processes, such as furniture manufacturing, joinery, cabinetry, packaging, and industrial housing construction [19]. Despite the presence of numerous literature reviews exploring the concepts of circularity and remanufacturing, low attention has been directed toward wood as a raw material and the remanufacturing potential of wood-based products. This represents a significant gap in the existing body of research.

For the wood products industry, the increased interest in circularity is evident, particularly in the European context. Organizations like the European Furniture Industries Confederation (EFIC) and the European Environmental Bureau (EEB) acknowledge the challenges and propose actions for circularity, emphasizing modern technologies, infrastructure, and circular business models [[20], [21], [22]]. The European Environmental Bureau (EEB) [20] underscores the multifaceted benefits achievable through circular economy interventions in the European furniture industry, such as value recovery, economic growth, and job creation while minimizing the resource consumption and the saving the environment. Specifically, the EEB estimates a potential gross value added of 4.9 billion euros, the creation of 160,000 new jobs, and a reduction in CO_2_ emissions by 3.3–5.7 million tons.

Challenges include the complex interplay between design, materials, business models, limited material availability, higher production costs, and regulatory gaps [22]. For reaching circularity at the customer side a societal and behavioral change is required, accompanied by the harmonization of circular economy regulations at the European level. Furthermore, challenges manifest on several fronts, including weak demand for recycled materials and second-hand furniture, poor design and quality of recycled material and products, poor consumer information, and limited reverse logistics infrastructure (i.e., product take-back systems) as well as the lack of competence regarding circular economy within the furniture industry [20].

For wood products, the raw material supply represents a significant environmental burden, as indicated by various studies [[23], [24], [25]]. To mitigate this environmental impact, circular practices, such as reuse and refurbishment, are commonly adopted. However, in cases where extensive renovation is required, material recycling or incineration is mainly chosen. As an example, only about 10 % of European furniture waste is recycled, with the majority being relegated to landfills or incineration [25]. In countries where biomaterial is extensively utilized for heat and energy generation, most of the wood raw material is incinerated. In Sweden, for instance, only 0.8 % goes to landfill. Remanufacturing, while still niche, is gaining demand from both public and private consumers emphasizing sustainable and circular practices. Public sectors, in particular, wield considerable influence as significant consumers of such products. Many are already reevaluating their business models to align with circular principles, underpinning a broader shift towards sustainability [26]. Laws and regulations, including European-level product passports, are expected to accelerate circular development [27]. Despite companies recognizing the potential of remanufacturing, they lack effective strategies to meet this demand. Challenges like underdeveloped logistics chains, suboptimal product design, and profitability difficulties hinder effective integration [28,29].

The paper aims to address the gap in remanufacturing research within the European wood products industry by exploring the current literature within the topic. Notably, recent studies by Mhatre et al. [18] and Vanacore et al. [25] highlight the limited research attention dedicated to remanufacturing in the context of furniture and other wood products. Hence, an evident research gap exists, necessitating further exploration of remanufacturing and its effective integration within the European wood products industry.

The primary objective of this paper is to establish a research agenda for remanufacturing practices within the wood products industry in Europe through a structured literature review. The results presented provide valuable knowledge that can guide future efforts aimed at rendering remanufacturing a practical and scalable endeavor within the European wood products industry.

The paper's structure includes an overview of key terms and theories on circular strategies and remanufacturing (Section 2) serving as the theoretical foundation of the study; a description of the research methodology (Section 3); content and thematic analyses of main findings (Sections 4, 5); the proposed research agenda (Section 6); and concluding managerial and research implications (Section 7).

## 2 Circular strategies and remanufacturing

**Fig. 1:**
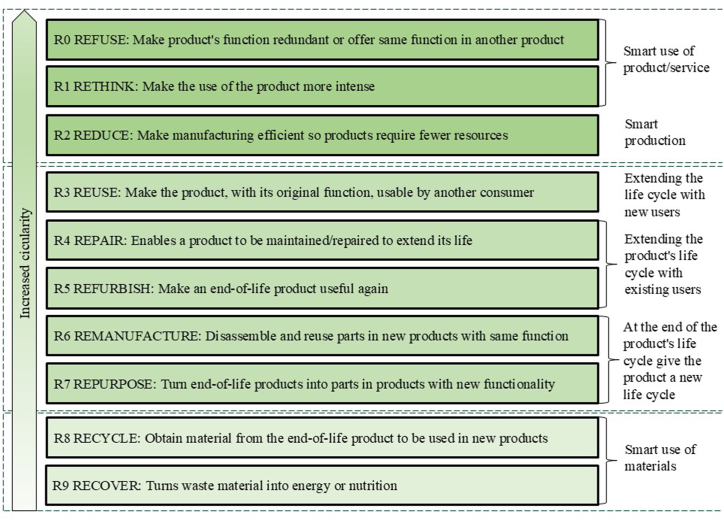
Circular strategies according to the 10R framework, adapted from Potting et al., 2017.

The description of circularity has evolved from a few activities, such as the 3R model *Reduce, Reuse, and Recycle*, to include many strategies to achieve circularity [30]. Today, the 10R framework is commonly used for describing the term [[31], [32], [33]]. The framework includes ten circular strategies, from taking care of product material in the end-of-life (EOL) to completely abstaining from a product. In the framework, these are referred to as (from top to bottom): Refuse, Rethink, Reduce, Reuse, Repair, Refurbish, Remanufacture, Repurpose, Recycle, and Recover, see Fig. 1. The top three strategies are about minimizing the efforts needed to be able to achieve a certain function connected to a product. This may mean minimizing production resources or sharing the product's function with several users, for example through leasing and rental agreements, or functional sales. The next five strategies aim to give the product one or more new life cycles, or to extend the product life cycle. This is where the "traditional" circular activities such as maintenance, repair, restoration, resale, and remanufacturing are to be found. In these strategies, it is assumed that the product can be reused or repaired with maintained or improved functionality. The two last strategies focus on efficient material use for EOL products. In these strategies, it is assumed that the product cannot be reused or repaired; only the material can be salvaged.

Remanufacturing is one of two strategies focusing on giving the product one or more new life cycles at the end of the life. While repurposing includes reusing the material and parts in new products with other functionality, remanufacturing is about regaining the same abilities and functionality as in the original product. The goal with remanufacturing is to achieve production that is economically and environmentally preferrable to new production [34,35]. The distinction between refurbishing, remanufacturing, and repurposing is somewhat unclear in the remanufacturing literature [1,32]. Some researchers argue that repairing a product on an industrial scale is to be seen as manufacturing. Others claim that a product consisting of old as well as new parts is to be seen as a modification or upgrade. British Standard Institute define remanufacturing as the process of “returning a used product to at least its original performance with a warranty that is equivalent or better than that of the newly manufactured product” [36]. Two important characteristics of remanufacturing, based on this definition, are that a remanufactured product must fulfill the same, or increased, functional requirements as a newly manufactured product, and you must be able to warrant the remanufactured product's performance to be “as good as new”. In the more recent standard EN 45553:2020 remanufacturing is defined as “an industrial process which produces a product from used products or used parts where at least one change is made which influences the safety, original performance, purpose or type of the product.” [37]. This definition highlights the industrial context of remanufacturing, emphasizing that the process involves making at least one modification to the product, transforming it to the extent that it may be regarded as a new product.

According to Otieno et al. [38], remanufacturing covers the following processes: acquisition of used products, reverse logistics, disassembly, cleaning, storage, rework, assembly, and testing. Research in remanufacturing mainly focuses on these processes [1] and includes models for disassembly, upgrading, renovation and reuse [6]. In later years, emphasis has been put on design and development (eco-design) as a prerequisite for effective remanufacturing [1].

Size-wise, remanufacturing is quite a large business. In the US, for instance, it employs about 430,000 employees in 73,000 companies and is worth $50 Billion [39]. Corresponding figures for the European market are 192,000 employees in 7204 companies and a turnover of around 30 billion euros [40]. Still, there is an enormous potential for increased business. The remanufacturing intensity, i.e., the ratio of remanufacturing to new manufacturing, is around 2 % in US and Europe (20, 28]. In the EU, the intensity varies from 11.5 % in aerospace industry to 0.3 % in maritime industry, with a remanufacturing intensity of the furniture industry of 0.4 % [40]. Increasing the remanufacturing intensity from 0.4 % to 2 % in the furniture industry, representing a market of 84 billion euro (EEB), represents a potential growth of around 1.5 billion euros.

Several studies have demonstrated the positive effects of remanufacturing, in firsthand as cost savings, energy savings, and increased material efficiency [15,34,35,41,42]. According to Liu et al. [2], remanufacturing can achieve significant savings compared to new manufacturing; the savings amount to 50 % in terms of costs, 60 % in terms of energy, 70 % in terms of materials and 80 % less air pollution. Other studies report on up to 83 % less energy consumed, 98 % material reduction, and 99 % less green gas emissions [1].

According to Gutowski et al. [39], three primary requirements for remanufacturing are: 1) that the products have significant residual value at the point of retiral, 2) that remanufacturing companies can effectively capture the retired product, and 3) that the products can be restored to a function that is as good as new with modest investments. Guide [43] lists seven characteristics of remanufacturing that significantly complicate production planning and follow-up: 1) uncertainty regarding when and how much return the remanufacturing provides, 2) the need to balance return against demand, 3) disassembly of products, 4) the uncertainty of materials being recycled, 5) the requirement for a reverse logistics network, 6) material matching limitations, as well as 7) wide variation in logistics and processing times.

Remanufacturing causes a dilemma for original equipment manufacturers (OEM), as the remanufactured products compete with new products. To deal with this dilemma, OEMs could apply different strategies [39]. An offensive strategy is to make it a part of the own business strategy, either by handling the remanufacturing internally or by partnering with a remanufacturing company. Other strategies are defensive, such as making used products inoperable, constant minor design changes, or buying back spent products. The high variability of the remanufacturing operations makes it difficult to apply traditional manufacturing techniques and strategies [2,5,38,44]. Diallo et al. [6], for instance, express a need for further research in the modeling of dismantling activities, yields and their impact on refurbishment and upgrading operations. Rizova et al. [5] recognize that existent decision-making models for manufacturing are not applicable for remanufacturing, and that decision-making models must be developed considering the uncertainties related to remanufacturing. The material salvage and disassembly possibilities are affected by product design, especially in terms of material properties (raw material but also coating, paint and joining material) and assembly techniques [1,2,38,45]. Accessibility to remanufacturable parts and materials depends on the customers' willingness to return the product at its EOL [46]. In this respect, business models based on leasing or renting could be a solution, as the product ownership remains with the seller [23]. Salah et al. [1] and Okorie et al. [7] highlight the need for reducing global and national barriers with international and government regulations and policies. Extended Producer responsibility (EPR) is an approach in which producers are given responsibility for the end-of-use treatment or disposal of products during the end-of-life. The responsibility is financial as well as technical, and the aim is to minimize the environmental impact of waste materials [47].

To succeed with remanufacturing, the integration of business models and design approaches and new forms of collaboration are required [3,7,20,28]. Jensen et al. [28] call for an integrated perspective on remanufacturing that includes product design and development, manufacturing and remanufacturing, and marketing and customer relationships when designing and managing the value throughout the value chain. The customer perspective has a major effect on the success of remanufacturing [8]. It can be difficult to meet customers' quality requirements for refurbished products, which is a major bottleneck for large-scale development of the remanufacturing industry [2]. Liu et al. [2] advocates a holistic systems perspective and intelligent automation solutions to overcome these problems. The positive impact of Industry 4.0 technologies is also highlighted by Kerin and Pham [3] and Salah et al. (2021) [1].

## 3 Research methodology

The increasing focus on remanufacturing of wood products and the lack of studies in this topic have prompted the authors’ interest in conducting this review. A systematic literature review was chosen as it enables the authors to explore the current theories and knowledge regarding remanufacturing of wood products in relevant literature.

### 3.1 Systematic literature review

The systematic literature review in this study followed steps adapted from the well-established methodology suggested by Tranfield et al. [48]: (a) defining the review's objective, (b) selecting appropriate databases and keywords, (c) screening and selecting abstracts and papers for data collection, (d) coding and analyzing the data, and (e) presenting the results deriving from the systematic literature review. The authors used the PRISMA checklist [49] as support for defining the purpose of the review, the methods used, and the findings obtained.

The primary objective of the systematic literature review was to establish a research agenda for remanufacturing practices within the wood products industry in Europe. The systematic literature review aimed at identifying and analyzing existing research within remanufacturing with a focus on the wood product industry.

The search was conducted between December 2022 and November 2023 in the scientific databases Scopus and Web of Science (see flowchart in Fig. 1). The search included terms “remanufacturing”, and “wood” or “furniture” in either title, abstract or keywords. The search included a combination of the keywords and searches with same search string was conducted in both Scopus and Web of Science. In the search results, peer-reviewed articles, conference papers, dissertations and theses were included. Only material using English as language and publication year between 1990 and 2023 were included. For these identified publications, the title and abstract were screened. Duplicates and publications not utilizing a combination of the keywords were excluded. Relevant hits without duplications were 37, of which 22 were peer-reviewed articles.

To expand the review sample, the 37 publications were reviewed for additional references that met the search criteria. A supplementary search in Google Scholar was also conducted using the same combination of keywords as the first search. The search resulted in over 3000 hits for “wood” and almost 6000 for “furniture”, whereof the title and abstracts of the first 100 of each were screened. Based on this, seven peer-reviewed articles and three conference papers were added to the list.

The 47 full publications were retrieved and reviewed by the authors of this article. During the review process, it became apparent that within the domain of the wood product industry or for wood material, the term "remanufacturing" carried dual interpretations. 38 publications adopted the same definition as used in this article, i.e., activities in the later phases of a product's lifecycle [1,3]. The alternative interpretation pertained to all additional processing of wood post-sawmill treatment, with synonymous terms like "secondary manufacturing" or "value-added manufacturing" [50]. Nine publications used this definition of remanufacturing and were therefore excluded. Publications that were not peer reviewed were also excluded in the content analysis.

20 peer-reviewed articles were selected and examined in three steps. The first step was to summarize and present descriptive data about the publications including the year of publication, source, research field. The second step was to conduct a qualitative inductive content analysis to structure and synthesize the content of the review sample. The 20 publications in the review sample were examined, and relevant data were extracted and documented.

After the systematic literature review, a supplementary search focusing on the European context were made using the keyword “remanufacturing” in the larger national research financiers’ project databases of Sweden and in the EU project database Cordis. This resulted in a list of 19 Swedish and 64 European projects on the topic of remanufacturing. None of the projects were targeting the wood and furniture industry in specific. The search was therefore broadened to include circularity in general, resulting in six Swedish and four European initiatives targeting the furniture and wood products industry. These publications together with the result from the literature review were used when the research agenda was developed.

A descriptive analysis was conducted to get a first understanding of the data set. Thereafter, an inductive approach was applied for the coding and analysis of the data [51]. As the study is explorative, an inductive content analysis was chosen to identify patterns and categories that have not been considered in previous literature. An inductive content analysis also encourages the authors to explore new perspectives that lead to new knowledge in the topic as well as increase the reliability of the study as the result is based on the identified data [51,52]. In the inductive content analysis, three categories were identified: 1) methodological approaches, 2) items of investigation, and 3) topics covered. The last step in the examination included a thematic analysis. In the thematic analysis influential factors for effective remanufacturing practices were identified. see for example [51]. This analysis was conducted to identify, analyze, and present themes. Systems theory was applied to categorize the recurring patterns [53], and three themes were identified: systems perspective, customer perspective, and manufacturing perspective. The research agenda was thereafter developed based on the review findings. The agenda conceptualizes and summarizes the findings.

### 3.2 Descriptive results

**Fig. 2:**
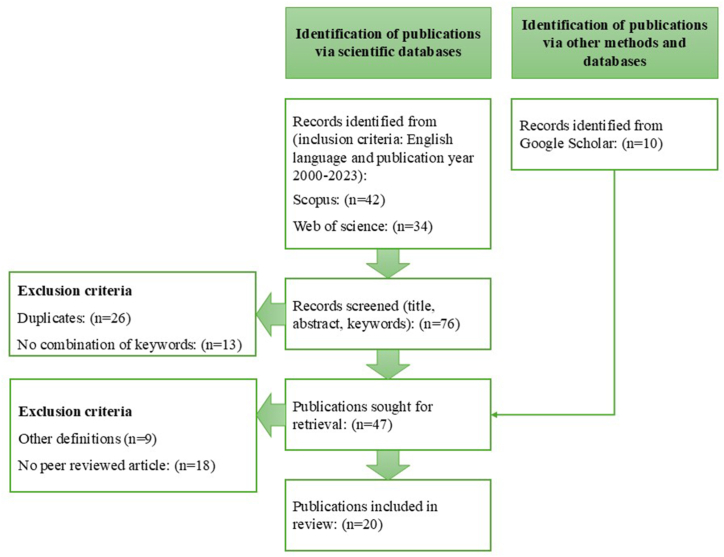
Flowchart of the systematic literature review.

**Fig. 3:**
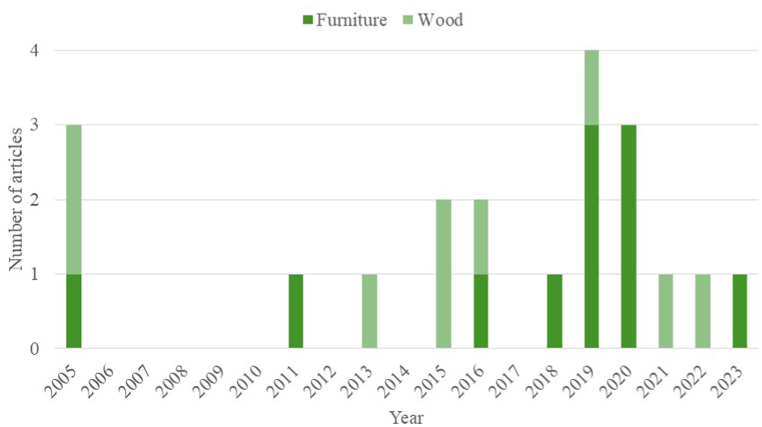
Year of publication.

The publication spans from 2005 to the current year, with a focus on the past decade, see Fig. 2. This trend is seen for articles on the topic of wood remanufacturing, but especially for furniture remanufacturing (see Fig. 3).

The articles are published in fifteen different journals, see Table 1. Two journals are recurring: Journal of Cleaner Production and Forest Products Journal. The journals cover three main topics: 1) wood science and wood products, 2) industrial and production engineering, and 3) sustainability.

**Table 1:** Journal frequency.

Journal title	Number of articles
Advances in Production Engineering & Management	1
Biofuels bioproducts & biorefining	1
Ciencia y Tecnologia	1
Environmental Science & Technology	1
Forest Products Journal	3
Frontiers in Sustainability	1
Indoor & Built Environment	1
Journal of Cleaner Production	5
Journal of Hazardous Materials	1
Journal of Remanufacturing	1
Mathematical Problems in Engineering	1
Production Planning & Control	1
Resources, Conservation & Recycling	1
Sustainability	1
Sustainable Cities & Society	1

Seven out of the 20 articles originated in the USA, while the rest of the articles are geographically spread in Europe, Asia, and North/South America. Two publications from USA [54,55], are authored by the same research group consisting of Falk, from the USDA Forest Service Forest Products Laboratory in Madison, Janowiak from Penn State University, and Cosper and Drozdz from the U-S- Army Construction Engineering Research Lab. Another two publications [56,57], from USA have Alanya-Rosenbaum as first author. Both are coauthored by Bergman and Gething, and with Mousavi-Avval as fourth author for [57]. Alyana-Rosenbaum, Bergman and Mousavi-Avval belong to the USDA Forest Service Forest Products Laboratory. Gething is affiliated with the National Wooden Pallet & Container Association in Alexandria. The three publications from Canada [24,58,59], are all authored by Cobut, Beaguegard and Blanchet from the Université Laval in Québec. The other publications from the USA and the rest of the world are all authored by different researchers and research groups.

## 4 Content analysis

Three main categories were identified through content analysis. The first category encompasses methodological approaches and data gathering methods, the second category encompasses items of investigation, and the third category encompasses primary topics covered in the articles.

### 4.1 Methodological approaches

Table 2 summarizes the *methodological approaches* in the articles. Quantitative studies, using experimental, case, and other empirical data sets for establishing and validating mathematical models, multi-criteria decision models (MCDM), and life cycle analyses (LCA), are predominant. MCDM is a generic decision making model commonly used for comparing different alternatives, while LCA is a method for assessing environmental impact of a product or system. Pure qualitative studies are less, mostly in the form of single and multiple case studies. Empirical studies use various data collection methods, such as surveys, interviews, field studies, and experiments.

**Table 2:** Methodological approaches covered.

Publication	Method						
	Theoretical				Empirical		Secondary data (4)
	Mathematical modelling (2)	MCDM (2)	LCA (5)	Other conceptual (1)	Case study (6)	Other empirical (10)	
Alanya-Rosenbaum et al. (2021)			x			x	x
Alanya-Rosenbaum et al. (2022)			x			x	x
Besch (2005)				x		x	
Cobut et al. (2015a)			x			x	
Cobut et al. (2015b)			x			x	
Cobut et al. (2016)			x			x	
Falk et al. (2005)						x	
Gutowski et al. (2011)							x
Janowiak et al. (2005)						x	
Jensen et al. (2019)					x		
Krystofik et al. (2018)					x		
Lin et al. (2020)	x					x	
Lingegård and von Oelreich (2023)					x		
Lindkvist Haziri et al. (2019)					x		
Ocampo et al. (2019)		x			x		
Otieno et al. (2020)		x					x
Sustanty et al. (2020)						x	
Suthar et al. (2016)					x		
Tornese et al. (2019)	x						
Yu (2013)^a^							

a Editorial.

### 4.2 Items of investigation and topics covered

Table 3 summarizes the *items of investigation* and *topics covered*. We In the inductive content analysis, four items of investigation were identified: 1) furniture, 2) wooden pallets, 3) interior products such as doors and windows, and 4) construction materials and building products. Eleven articles out of 20 address furniture either as the main item of investigation, or as one of several items. Eight of the articles address office furniture in specific; five as main item of investigation [23,34,38,42,60], and three as one item in multiple case studies [28,29,39]. Three articles address wooden furniture waste recycling and reverse logistics [[61], [62], [63]]. Remanufacturing of wood pallets were investigated in three articles: [56,57,64]. Interior wood products for nonresidential applications were studied in Refs. [24,58,59], and doors specifically in Ref. [28]. Falk et al. [54] and Janowiak et al. [55] studied construction material, while Yu (2013) discusses reuse of construction material and building products.

**Table 3:** Items of investigation and topics covered.

Publication	Item(s) of investigation	Topics	Key findings
Suthar et al. (2016)	Furniture (Other items: recycled materials such as paper, glass, and plastic)	Waste recycling and waste management	Investigates the role of different stakeholders in an Indian waste recycling case study. The findings show that remanufacturing industry acts as main buyer of the recycled products, and that informal waste recycling systems creates business opportunities for urban people.
Susanty et al. (2020)	Furniture	Waste recycling and waste management, Reverse logistics	Investigates the effect of supply chain cooperation on circular economy practices. Findings shows that customer collaborations improve the economic and environmental performance while supplier collaboration improves the environmental performance.
Ocampo et al. (2019)	Furniture	Waste recycling and waste management, Reverse logistics, Decision models for remanufacturing	Proposes a framework for location planning of collection and distribution centers for reverse logistics using a MCDM approach. Government policies and regulations largely affects the facility location decision, as well as the quantity and quality of retaken products.
Lin et al. (2020)	Furniture	Waste recycling and waste management, Decision models for remanufacturing	Proposes a cradle-to-cradle programming model for profit maximization of circular strategies for furniture based on the 3R model. Public compulsory procurement, subsidies, and corporate social responsibility are seen as important measures to reach improved environmental impact.
Falk et al. (2005)	Construction material	Waste recycling and waste management	Investigates the worker and workplace exposure of lead during wood remilling operations. The findings indicate that the operations are safe if appropriate equipment is used.
Janowiak et al. (2005)	Construction material	Waste recycling and waste management	Investigates the possibilities to recycle wood material from US military buildings. The findings indicate that especially tongue & grooving flooring is suitable for remanufacturing, and up to 50 % of the recycled material can be reused.
Alanya-Rosenbaum et al. (2021)	Wood pallets	Life cycle analysis	Presents a cradle-to-grave environmental analysis of wood pallet manufacturing and recycling using data from the US. The highest impact was found in the manufacturing and raw material supply stages, while repair activities were identified as important mitigators, as they extend the service life of the pallets.
Alanya-Rosenbaum et al. (2022)	Wood pallets	Life cycle analysis, Remanufacturing production system	Studies the life cycle impact of repair and remanufacturing of wood pallets based on data from facilities in the US. The findings identified transportation, electricity consumption during remanufacturing, and fastening material as most impactful.
Gutowski et al. (2011)	Furniture (Other items: clothing, computers, electric motors, tires, appliances, engines, and toner cartridges)	Life cycle analysis	Studies the energy saving potential of remanufacturing using data from 25 case studies. Findings show that the energy savings are large for products such as furniture, clothing, and computer equipment, while remanufacturing of tires, computers, monitors, and household appliances results in significant added energy consumption.
Krystofik et al. (2018)	Furniture	Life cycle analysis	Studies the life cycle effect of multiple remanufacturing cycles of office furniture and proposes an adaptive remanufacturing approach where remanufacturing is combined with customizations and modifications of the product to keep it up to date during its lifecycle.
Cobut et al. (2015a)	Interior products	Life cycle analysis	Presents a cradle-to-grave environmental analysis of interior wood doors in a North American context. Results indicate that production of raw materials and end-of-life transportation give rise to the highest environmental impact. This is Part 1 of a two part publication.
Cobut et al. (2015b)	Interior products	Eco-design	Proposes an eco-design strategy for interior wood doors. The most promising scenario was the recovery and remanufacturing of doors, and developing services for this was suggested. This is Part 2 of a two part publication.
Cobut et al. (2016)	Interior products	Eco-design	Describes environmental profiles for appearance wood products, which could be used for developing eco-design solutions. Type of material and product weight are the most impactful variables. Remanufacturing and reclaiming used products were suggested for solid wood.
Lindkvist Haziri et al. (2019)	Furniture (Other items: food processing machines and material handling machines)	Eco-design	Studies the integration of feedback from different life cycle phases into the product design. Five barriers (lack of internal awareness, knowledge, incentives, feedback channels, and supportive structures) and five enablers (integrated design processes, customer demand, laws, regulations and standards, and emerging technologies) were found.
Yu (2013)	Construction material	Eco-design	In this editorial article, the author highlights the economic and environmental benefits of reusing and remanufacturing construction material in terms of cost savings and reduced landfill, use of virgin material, and CO_2_ emissions.
Tornese et al. (2019)	Wood pallets	Reverse logistics	Studies the reverse logistics of wooden pallet and the use of preventive maintenance schedules, referred to preemptive repair. The results show that preemptive repair has both economic and environmental benefits.
Otieno et al. (2020)	Furniture	Decision models for remanufacturing	Proposes a framework for remanufacturability of office furniture using a fuzzy TOPSIS and MCDM approach. The methodology assesses the economic, environmental, and social aspects of remanufacturability.
Besch (2005)	Furniture	Product-service systems and business innovation	Describes opportunities and barriers for the implementation of Product-service systems for office furniture on the European furniture market. The author concludes that, for successful implementation, factors such as profitability, organization, market demand, and the packaging of the service should be considered.
Jensen et al. (2019)	Furniture (Other items: wind turbines and healthcare equipment)	Product-service systems and business innovation	Proposes an integrated perspective on remanufacturing for driving sustainable value creation. Sustainability related factors and integrating mechanisms are identified in a multiple case study.
Lingegård and von Oelreich (2023)	Furniture	Product-service systems and business innovation	Studies circular public procurement implementation and management in a case study setting. The findings indicate that centralized government, standardization, follow-up, and inventory systems are crucial factors for succeeding with circular public procurement.

During the inductive content analysis, topics covered in the literature were identified as adjacent to or related to remanufacturing. These topics are presented in Table 3 under the column “Topics”. The most common topics discussed in combination with remanufacturing were waste recycling and waste management and life cycle analysis (LCA). Other related topics were eco-design, reverse logistics, decision models and product service systems (PSS). Only one source covered the topic of remanufacturing production systems. Some articles addressed two or more topics. The topics are further elaborated in the following part of this study.

#### 4.2.1 Waste recycling and waste management

Waste recycling and trading in India was investigated by Suthar et al. [61] in the form of a case study conducted in Dehradun, the capital of Uttarakhand. Wooden furniture was mainly collected for secondhand trading while wooden scrap was reused for building construction, while other products and material were supplied to the remanufacturing industry. Furniture waste recycling in the remanufacturing supply chain was also addressed in Refs. [42,62,63]. The possibility to reuse construction wood in the form of military barracks was studied in Refs. [54,55].

#### 4.2.2 Life cycle analysis

Life-cycle analysis (LCA) was emphasized in five of the publications. The items of investigation were wood pallets, interior products, and furniture. Both articles by the research group Alanya- Rosenbaum et al. performed LCA or LCIA (Life-cycle-impact-analysis) of wood pallet repair and remanufacturing sector in the United States [56,57]. According to Alanya-Rosenbaum et al. [57] wood pallets are ubiquitous products that can be recovered and reused. The scope of the LCA included the life-cycle stages from forest resource activities to end of life for wood pallets. Cobut et al. [24] conducted an LCA of interior wood doors used in non-residential buildings. Primary data was obtained from one manufacturer of wood doors in Quebec, Canada. Krystofik et al. [34] conducted a comparative LCA analysis to estimate the impacts of multiple remanufacturing cycles of office furniture products. The data was obtained from a remanufacturer. In Ref. [39] the impact of remanufacturing on energy consumption was investigated in 25 cases including eight different product categories, of which furniture was one. Energy savings were noted for furniture, textile, and computer equipment, while remanufacturing of refrigerators, washing machines, and computers had a negative impact, implying that buying a new product is better.

#### 4.2.3 Eco-design

Cobut et al. [58] explored different scenarios of eco-design. This study was based on results from a study of LCA of interior wood doors for non-residential buildings [24]. The life cycles stages included Raw materials, Transportation and End-of –life. The original scenario for End-of-life for interior wood doors was landfilling, and as an alternative to landfilling, remanufacturing/reutilization/recycling of the door core assembly was proposed. The scenario includes a description of how the door core can be reutilized into a new door. Results from this study show that extending the life of the core assembly has a strong beneficial impact on the ecosystem. Moreover [59], suggested solutions for the eco-design of interior appearance wood products used in nonresidential applications, based on the studies presented in Refs. [24,58]. The wood products in this study included, amongst other products, office desks. Cobut et al. [59] suggested that remanufacturing could be a reasonable end-of-life option for office desks. Lindkvist Haziri et al. [29] conducted a multi case study of barriers and enablers for effective information feedback from remanufacturing into the product design. Three cases were included, whereof an office furniture company was one. The findings regarding barriers were lack of awareness, knowledge, incentives, feedback channels, and supporting organizational structures while business opportunities, integrated design processes, customer demand, regulations, and innovative technologies were seen as enablers. Yu [65] promotes the remanufacture and reuse of treated construction material and other building products that reached end of life and recognize modern construction methods as a means for eco design of houses and building, as they enable e.g., easy deconstruction and disassembly.

#### 4.2.4 Reverse logistics

In [62], a multi-criteria decision-making approach was developed for location planning in reverse logistics. The approach was tested in a case study of a furniture firm in Ceby, Philippines. The linkage between circular economy practices and the environmental and economic performance was investigated in Ref. [63] by the means of a questionnaire study directed to wood furniture Small and Medium Sized Enterprises (SME) in Indonesia. The study focused on environmental-oriented supply chain cooperation practices, and the results showed that the SMEs could be divided into leaders, followers, and laggards, with respect to these practices. Tornese et al. [64] studied the economic and environmental impacts on reverse logistics when introducing a preventive remanufacturing strategy of wooden pallets. This was achieved by developing an integer linear optimization model.

#### 4.2.5 Decision models for remanufacturing

A mathematical modelling approach using particle swarm optimization was proposed in Ref. [42] for the evaluation of the total profit on recycled waste furniture. The approach was tested using simulations based partly on empirical data from the Taiwanese waste recycling system. Ocampo et al. [62] developed a multi-criteria decision-making (MCDM) approach for collection and distribution center location decisions. The applicability of the approach was tested in a case study in the Philippines. Otieno et al. [38] developed a MCDM approach for evaluating the manufacturability of office furniture based on economic, environmental, and social criteria. The approach was tested using secondary case data regarding three types of products.

#### 4.2.6 Product-service systems and business innovation

Besch [23] proposed a product-service system approach for office furniture and tested the idea with 30 European office furniture producers, customers, and experts in the area. The data was collected through telephone interviews. It proved difficult to realize the concept under prevailing market conditions. Jensen et al. [28] explored in three case studies whether an integrated perspective of remanufacturing could drive sustainable value creation. They found evidence of impact on the triple-bottom-line but noted that investments in remanufacturing were mainly driven by profits. Lingegård and von Oelreich [60] studied public procurement of furniture in the public sector was studied in and elaborate on how PSS can be used in the public sector. An important finding is that the prerequisites of the procurer organization impact the implementation of circular public procurement (CPP). Centralized management and government of CPP, implementing a standard assortment of furniture, the use of use-based or performance-based PSS, and a system that supports the management of circular flows are suggested factors for improving CPP implementation.

#### 4.2.7 Remanufacturing production system

There exist a limited number of articles focusing on remanufacturing production systems. In our review, only one article described the remanufacturing processes in production [57]; described a wood pallet repair and remanufacturing system. According to Ref. [57], the repair and remanufacturing stages comprise three processes: 1) dismantling or board preparation, 2) pallet assembly or repair 3) painting and or stamping.

## 5 Thematic analysis

Influential factors for effective remanufacturing practices were identified in the thematic analysis. Each factor was categorized into one or more of three perspectives inspired by systems theory [53]: systems perspective, customer perspective, and manufacturer perspective. In this article, the system's perspective includes aspects on a systemic level that affect more than one actor. Customer perspective focuses on aspects that derive at the customer or affect a customer's behavior. Manufacturer perspective manage aspects based on the product design, and the product realization process, and influence the manufacturers' behavior.

### 5.1 Systems perspective

Collaboration between actors in the value chain as well as suitable reverse logistic systems are identified as essential for remanufacturing practices [28,34,56,57,59,63]. According to the findings of Susanty et al. [63], upstream collaboration can improve the eco-design practices and the environmental performance, while downstream collaboration has positive impacts on both the internal environmental management and eco-design practices, and thus, contributes positively to the environmental as well as economic performance. Customer collaboration, especially in co-design and establishing waste collection systems, is therefore an enabler for successful remanufacturing practices. Moreover, Jensen et al. [28] identified the downstream collaboration with dealerships as a challenge, as dealers maintain the relationship with customers. Consequently, collaboration between actors in the value chain, both upstream and downstream, is considered as an enabling factor for effective remanufacturing practices.

Most furniture ends up in landfill; between 80 and 90 % of furniture waste is incinerated or sent to landfill, and only 10 % of furniture waste is recycled [25,59]. As a comparison, only 0.8 % of the furniture waste goes to the landfill Sweden, while the majority is incinerated (recovered as energy). According to Lin et al. [42], the situation of Taiwan is similar, and the authors suggests three different strategies for improving the recycling of furniture waste: compulsory procurement of remanufactured furniture by the authorities, providing subsidies to encourage the recycling of waste furniture, and increased purchase of remanufactured furniture by top firms in Taiwan. These three strategies are considered as enablers for remanufacturing practices as they lead to increased profits, decreased CO_2_ emissions, and increased reuse of waste.

According to Suthar et al. [61], who studied the waste trading business of urban materials in India, informal waste recycling is one source of raw material supply for remanufacturing. The remanufacturing industry's locality seemed to determine the profitability of informal waste recycling. This is confirmed by other researchers as well. For example, Ocampo et al. [62] developed a multi criteria decision making approach for the location of collection and distribution centers in the Philippines. The findings implicate that proximity to customer is a critical aspect for locating distribution centers for remanufactured products, while for the collection centers, the location decision was influenced by government policies and regulations as well as material availability and supply of product return. In the interview study of Besch [23], the distance between the service provider and the customer was seen as highly affecting the possible implementation of product-service systems (PSS) for office furniture. Based on this, the locality of remanufacturing industry and closeness to actors in the value chain are factors influencing remanufacturing. In addition, current waste management practices, government policies, and regulations can be considered as factors influencing remanufacturing practices.

### 5.2 Customer perspective

Challenging factors identified in literature regarding customers are price sensitivity and product branding/fashion [23,28]. Customers might also perceive the rental or remanufactured furniture as something bad, which could damage the customers’ reputation. Both Besch [23] and Jensen et al. [28] point out the extrinsic value of office furniture as an important aspect of consideration. Fashion and design choices lead to premature replacement of furniture. On the one hand, a well-planned supply of products for remanufacturing is an enabler, on the other hand it does not rhyme well with long-term rental contracts. To overcome this, according to Ref. [23], there is the possibility to include product modifying services as a part of the rental agreement. In the interview study reported in Ref. [29] an office furniture manufacturer reflects on the robustness of the products, in that they do not wear out, but rather becomes uglier. The changing design requirements from the customers is a challenge for the remanufacturers as they need to design and create products that look better than the original products, referred to as adaptive remanufacturing in Ref. [34]. Based on this, the customer perception of rental or remanufactured products as well as price sensitivity are factors influencing the remanufacturing practices. Lingegård and von Oelreich [60] focused on the public sector and barriers for implementing circular public procurement contracts for a university. The results of this study emphasize the agents of change in the public sector to implement contracts in the entire organization.

### 5.3 Manufacturer perspective

Manufacturers can develop business opportunities from remanufacturing. However, in previous studies, cannibalization of sales from new products is considered a challenge for remanufactured products. Cannibalization here means when the purchase of a remanufactured version of a product cuts the sale of a new product. This is for instance raised by an office furniture manufacturer in Ref. [29]. In that case, the manufacturer limited the sales of remanufactured furniture to the local customers to avoid cannibalism.

Previous studies state that the absence of a suitable remanufacturing business model for the manufacturers can be a challenge. In a case study of a UK furniture manufacturer reported in Ref. [28], the reason behind the inability to gain full value from eco-design investments was the absence of a suitable remanufacturing business model. This is also raised as a challenge in Ref. [23]; the European office furniture industry consists of SMEs with limited resources to develop new business models. To deal with this challenge, Besch [23] proposes using the concept of PSS to build sustainable remanufacturing business models and for dealing with the dilemma of cannibalism. Besch argues that product-oriented as well as use-oriented PSS are well suited for office furniture. The product-oriented PPS might be in the form of additional services such as maintenance and repair offered in combination with the product's sales. For the use-oriented PSS, the OEM might own the product during its lifetime and due to this, the product can be available to the customer through renting or leasing. In addition, manufacturers stated that the remanufacturing process would be the critical part of the PSS, as remanufacturing requires individualization of work tasks and a non-standard manufacturing process [23]. However, Besch argues that cost savings, for instance in the form of raw material and personnel that are major costs in furniture manufacturing, might partly cover the losses. Due to this, the absence of new business models for remanufacturing is considered a hindering factor for effective remanufacturing practices.

In [38], the number of joints highly affected the efficiency of remanufacturing. One furniture manufacturing company has successfully adapted the product design by lowering the part counts and quick manual disassembly/reassembly, and in this way minimized the replacement of parts during remanufacturing [28]. Cobut et al. [24,58] propose that the interior door core can be salvaged, while new faces are added during remanufacturing. Remanufacturing is from this perspective a matter of according to the review surfaces and fabrics. Adopting a preventive remanufacturing policy could be a way to reduce the total reverse logistics during the product life cycle [64]. The findings in Tornese et al. [64] indicate that preventively sending wooden pallets for remanufacturing, i.e., to repair or replace the pallets before a functional failure, has positive economic as well as environmental impact on reverse logistics. Krystofik et al. [34] recognizes the differences in business opportunities of OEM and third-party remanufacturers regarding access to a complete design and manufacturing process. The OEMs have an advantage, in that they drive product innovation and can dictate changes in the design for enabling remanufacturing, while the third-party remanufacturer has no control of the product design. This has also impacted on the possibility to apply PSS models, as they are firsthand coupled with OEM activities. Therefore, product design is a factor influencing remanufacturing practices.

Another factor influencing remanufacturing practices identified in literature is the variation in the supply of returned products. Possible ways to handle this problem are, according to Besch [23], to regulate the supply in rental contracts, or the use of third-party companies that handle the collection of products and the remanufacturing. Even if a reliable supply could be established, the variations in product types and quality is likely to be high [34]. Poor quality of reused products might increase the material use and cost in remanufacturing. For supporting the remanufacturing decision making, Otieno et al. [38] developed a method for evaluating the remanufacturing potential of different office furniture products. Their findings indicated that differences occur for cost aspects like acquisition, disassembly, replacement, and refinish, while function, energy savings, and reassembly cost were similar for all cases.

Remanufacturing might be a way to reduce energy consumption during the lifecycle of wood product, see for example [24,[56], [57], [58], [59]] due to the low or non-existent power consumption during the use phase [39]. In Ref. [42], remanufacturing of furniture contributed to reduced CO_2_ up to tenfold, depending on the strategy applied. Krystofik et al. [34] studied the environmental impacts of office furniture, in which the original manufacturing, adaptive manufacturing, and remanufacturing were compared with cumulative energy demand. The findings showed that the total environmental benefits increased with the number of remanufacturing cycles. Moreover, adaptive manufacturing increases the magnitude of benefits in a multi-cycle remanufacturing context, as it can lower the impact of subsequent remanufacturing cycle where an increased impact is normally expected. Due to this, the number of remanufacturing cycles is a factor influencing remanufacturing practices. For office furniture, the major production cost is raw material [23] and remanufacturing therefore significantly affects the total lifecycle cost of the product. The number of remanufacturing cycles is also an enabler for reducing the use of virgin wood material, in this case wood as it prolongs the use of raw materials.

### 5.4 Results summary

**Fig. 4:**
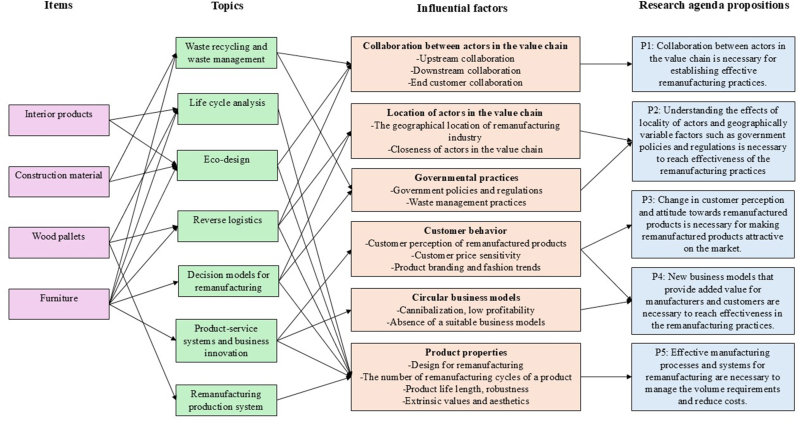
Conceptual framework of remanufacturing in the wood products industry.

The thematic analysis identified a number of factors that influence remanufacturing practices in the wood products industry. These factors are summarized in Fig. 4. The influential factors are also related with the items of investigation and topics extracted from the content analysis. Further research needs have been identified based on the content and thematic analysis. There needs are discussed in the next section and summarized into five proposals. The connections between influential factors and the proposals are included in the summary. Fig. 4, thus, serves as a conceptualization of remanufacturing in the wood products industry.

Research on interior products mainly regard LCA and eco-design aspects, while waste management and eco-design are topics related with construction material. Wood pallet research regards remanufacturing production systems and reverse logistics. Furniture was the most common item of investigation, and the research cover almost all topics. Common research gaps with respect to items or topics are hard to identify considering the low number of articles found. Nevertheless, looking at the topical coverage, the research is mainly generic or peripherical, and an evident research gap is found for remanufacturing from an operations or manufacturing perspective. In addition, strategic aspects in the form of decision support and business models have been studied considering furniture, leaving a research gap regarding strategic aspects for other types of products.

Following influential factors were identified in the thematic analysis: collaboration between actors in the supply chain, location of actors, governmental practices, customer behavior, product properties, and circular business models. *Collaboration* is related with upstream (waste management and eco-design) and downstream (waste recycling and reverse logistics) topics, pointing out the importance of collaboration with customers. *Location of actors* is important when designing a reverse logistics system, as the geographical location and relative closeness of actors affect the effectiveness. These factors, together with governmental practices, were seen as highly influencing factors to be considered in remanufacturing decisions. *Governmental practices* are also connected with waste management practices, as laws, regulations, and systems for waste management differs between regions and counties. *Customer behavior* is tightly connected with the topic of product-service systems and business innovation, as the customer design and brand choices, and price sensitivity affect the overall perception of remanufactured products. The topic of product-service systems and business innovation is naturally connected with *circular business models*, and the problems of creating profitable and suitable business models for remanufacturing. *Product properties* is an influential factor related with several topics. The remanufacturability of products is an eco-design aspect that determines the number of remanufacturing cycles, and which affects the remanufacturing production system and the reverse logistics system. Product life length and robustness are factors that affect the environmental and economic impact of remanufacturing, thus connected with life cycle assessment, decision support, and business innovation topics. Extrinsic values and aesthetics are factors that should be considered in business innovation as they affect customer behavior.

## 6 A research agenda

Based on the results of chapter 5, critical areas for further development of remanufacturing practices in the wood products industry in a European context have been identified.

From a *systems perspective*, collaboration was emphasized in literature as a key factor for successful remanufacturing within the wood products industry [28,34,56,57,59,63]. The findings show that both upstream and downstream collaboration is needed for establishing efficient and effective remanufacturing. Upstream, effective collaboration is needed for enabling sustainable material sourcing and selection and design for remanufacturing, i.e., products designed for efficient disassembly, repair, and reassembly. Thus, upstream collaboration is a prerequisite for eco-design.

Downstream collaboration is a prerequisite for the creation of efficient reverse logistics systems, and for influencing the customer's perception of remanufactured products. For supporting effective collaboration in the value chain, further research of cooperative mechanisms in dyadic or triadic stakeholder partnerships is needed. Research areas connected with this include how to reach effective collaboration between remanufacturers, suppliers, and designers for enabling eco design, as well as how to create effective collaboration between remanufacturers, dealers, and customers for establishing waste collection systems and for including the customer in the remanufacturing process.

Moreover, increased collaboration is a way to approach the traceability issue. If products cannot be tracked after they leave production, it is difficult to achieve efficient reverse logistics and secure product return flows. It can also be difficult to track usage patterns, which affects the ability to guarantee functionality. Research on information and knowledge management in general is needed, as well as studies and solutions towards specific topics, such as information requirements during separate phases of the product lifecycle(s) and coordinated information flows, development and utilization of information and communication technologies for information and knowledge sharing.

In addition, collaboration is a means of increasing the circular preparedness of the industry. The review findings only implicitly state this as a challenge for remanufacturing within the wood products industry, but the need for cooperation and communication between stakeholders at the industry level is in line with the anticipated need for circular development as stated by Refs. [2,20,28,66]. Increased collaboration has positive effects in the form of new relationships and business opportunities, which contributes to increased awareness of circular strategies and consensus at industry level. Based on this, following proposition is made.

P1 Collaboration between actors in the value chain is necessary for establishing effective remanufacturing practices.

The locality of different actors affects the effectiveness of the remanufacturing practices. In the literature review, the proximity to customers, collection facilities and distribution centers were seen as important allocation aspects [23,61,62]. Further research is required on how to make optimal allocation decisions with regards to the remanufacturing value chain and how these decisions are practically implemented, for instance in the form of local ecosystems or regional closed loop systems. Other geographically variable factors that affect the allocation decision are for instance government policies, subsidies, taxation, and land prices. Government policies and regulations on regional, national, and global level have been pointed out as a main factor of impact and challenge for circular strategies like remanufacturing. Therefore, increased understanding of how government policies and regulations affect location choice is another area of research. Following proposition reflects the further research directions.

P2 Understanding the effects of locality of actors and geographically variable factors such as government policies and regulations is necessary to reach effectiveness of the remanufacturing practices.

From the *customer perspective*, the main challenges in the review were related to customer behavior and attitudes, which is in accordance with findings related to remanufacturing in general, see e.g., Refs. [8,28]. Customers have few incentives to choose remanufactured products, and since wood products do not wear out like products with shorter life cycles, the product's extrinsic value affects the lifecycle and customer behavior to a greater extent. Further research is needed to understand customer attitudes and behavior and for finding ways to support customer preferences towards remanufactured products.

A research area related to proposition three regards the collaboration between the customer and the manufacturer, in that increased cooperation between the customer and manufacturer is a way to facilitate the customers' choice of remanufactured products to a greater extent, for example through co-design, sustainability-related key performance indicators that support procurement and follow-up, and business models enabling easy procurement and access to remanufactured products. Following proposition highlights the needs from the customer perspective.

P3 Change in customer perception and attitude towards remanufactured products is necessary for making remanufactured products attractive on the market.

According to the review, successful remanufacturing practices are enabled by the development of circular business models. From the *manufacturer perspective*, several directions were pointed out, such as the use of PSS and co-design approaches [23,28,29]. Use-oriented models in the form of leasing and renting are ways to handle the return logistics, while business models allowing design modifications during remanufacturing through co-design address the problem with customer perception and attitudes. Further research on how to develop suitable business models and contract forms is, therefore, required. This includes aspects of value proposition modelling, contract regulation, and marketing, but also how to build suitable internal capabilities supporting the business model. The characteristics of the wood products industry must be understood and reflected in the development of solutions [23,63]. The manufacturers are mainly SMEs with limited resources. In addition, all manufacturers are not OEM. Specific focus should therefore be given to the characteristics of SMEs and third-party manufacturers, and how to enable effective communication and cooperation between OEM and third-party manufacturers. Proposition four reflects the further research directions with regards to business modelling.

P4 New business models that provide added value for manufacturers and customers are necessary to reach effectiveness in the remanufacturing practices.

Further research is needed for developing suitable manufacturing processes, strategies, and systems [2,28,34,38]. At the process level, the coordination between manufacturing and remanufacturing, as well as combining automation and manual processes, may be means to achieve effectiveness. Coordinated planning and control, process optimization, and process related performance metrics are examples of areas of further studies. Another area of research regards design for remanufacturing, for instance regarding the number of parts to be disassembled and number of operations required [28,34,38]. At the manufacturing strategy level, competitive factors relate to various decisions affecting the production system. Correspondingly, the decisions made in production affect the competitive factors. Two factors directly impacting the design of a production system are volume and variety of products [23,34]. Achieving fully automated and centralized remanufacturing is difficult in the wood products industry because of the volume, variety, and quality fluctuations of the products. Highly specialized and flexible remanufacturing in turn entails high production costs. To achieve the necessary flexibility, digital tools and flexible automation solutions can be used. Following proposition is made for addressing the need of further research regarding manufacturing strategies.

P5 Effective manufacturing processes and systems for remanufacturing are necessary to manage the volume requirements and reduce costs.

## 7 Conclusions

This paper underscores the increasing attention drawn to the field of remanufacturing. Nonetheless, it is evident that research on remanufacturing in the wood products sector remains significantly underexplored. To address this deficiency, a comprehensive research agenda is proposed with the aim of accelerating much-needed research efforts in this domain. From the systems perspective, collaboration and cooperation are crucial, and developing solutions enabling collaboration is therefore a prioritized research area. Furthermore, a deeper understanding of customer perceptions and behavior, along with the formulation of economically viable remanufacturing strategies, are prerequisites for developing circular business models that give incentives and benefits for all stakeholders and positive systemic effects. In the future, circular products are the default choice for consumers, who should perceive remanufactured items as equal to or even superior to new ones. The pursuit of this paradigm shift requires interdisciplinary research to bridge the gap between current practices and the aspiration of a circular economy.

The proposed research agenda aligns with the identified imperatives for fostering circularity within the European wood products industry, as defined by the European Environmental Bureau [20,21], and the European Furniture Industries Confederation [22]. Above all, it addresses the pressing need for innovative infrastructure solutions and circular business models. It is also aligned with the suggested research agendas for remanufacturing presented by for instance Refs. [[1], [2], [3],[6], [7], [8]]. The shared emphasis on topics such as value chain mechanisms, collaboration, taxation, policies, legislation, production strategies, business models for remanufacturing, comprehension of customer perceptions and acceptance, and the principles of design for manufacturing underscores the comprehensive applicability of this research agenda. Hence it is likely that the research agenda has a general validity, with applicability not only within the European context but also on a global scale.

Various remanufacturing researchers, including [3,5,7], underscore the need for specific solutions, such as modeling techniques and emerging technologies. While the suggested research agenda does not explicitly tackle this aspect, the development of these solutions is crucial for fostering collaboration and informed decision-making in the wood products industry. In this context, digitalization and automation serve as key facilitators in realizing circular strategies.

## CRediT authorship contribution statement

**Mirka Kans:** Writing – original draft, Methodology, Investigation, Formal analysis, Conceptualization. **Malin Löfving:** Writing – original draft, Methodology, Investigation, Formal analysis, Conceptualization.

## Data availability statement

Data will be made available on request.

## Declaration of competing interest

The authors declare the following financial interests/personal relationships which may be considered as potential competing interests: Mirka Kans reports financial support was provided by Swedish Energy Agency. Malin Lofving reports financial support was provided by 10.13039/501100004527Swedish Energy Agency.
